# Belief in biological origin of race (racial essentialism) increases sensitivities to cultural category changes measured by ERP mismatch negativity (MMN)

**DOI:** 10.1038/s41598-022-08399-3

**Published:** 2022-03-15

**Authors:** Ginger Qinghong Zeng, Xue-Zhen Xiao, Yang Wang, Chun-Yu Tse

**Affiliations:** 1 Institute of Advanced Technology, University of Science and Technology of China, Hefei, China; 2 Department of Psychology, The Chinese University of Hong Kong, Hong Kong, China; 3 School of Humanities and Social Science, The Chinese University of Hong Kong, Shenzhen, China; 4 Department of Social and Behavioral Sciences, City University of Hong Kong, Hong Kong SAR, China

**Keywords:** Psychology, Cognitive neuroscience, Social neuroscience

## Abstract

The dynamic multicultural view explains culture-specific effects on cognition that cultural knowledge is organized in multiple knowledge systems which are specific to each culture and differentially activated when exposed to related objects or scenes. This view predicts automatic categorizations of environmental information according to the culture-specific knowledge systems. This study investigated whether cultural information could be spontaneously categorized, and the modulation of this process by the belief in the biological origin of race (i.e., racial essentialism) with an event-related brain potential, the visual Mismatch Negativity (vMMN). Deviant pictures of Eastern (Western) culture were randomly presented in a stream of standard Western (Eastern) pictures while participants were playing a video game. Participants who endorse racial essentialism (high group) showed vMMNs to the deviants with high relevance to the Eastern or Western culture and the deviant with low Eastern relevance; while participants with low racial essentialism showed vMMN to the deviant with high Eastern relevance only. These results revealed spontaneous cultural categorization with vMMN and the top-down modulation of spontaneous categorization by personal belief. In addition, this is the first demonstration of MMNs to cultural deviance and the potentials in applying MMNs to study psychological essentialism and social categorization.

## Introduction

Culture refers to the ideas and practices that are shared by a group of people and can be transmitted across generations^1^. Earlier theories suggested that the knowledge of all cultures is integrated in a single knowledge system that continuously influences cognition and behaviors independent of the cultural context. However, the dynamic multicultural view^2^ postulated that the knowledge of individual culture is organized in a specific knowledge system. When exposed to the objects or scenes associated with one’s own culture (i.e., cultural cues or primes), the knowledge system of that specific culture is activated and exerts culture-specific effects on cognition and behaviors^2^. Bicultural individuals who are more experienced and knowledgeable about their non-dominant culture were more readily primed with the non-dominant cultural cues than those who have less experience in the non-dominant culture^3^.

This culture-specific priming effect was found even when the cultural cues were only presented for 10 ms using a masked priming procedure^4^. For example, when presented with Chinese writings, Canadian Chinese were more likely to describe themselves with adjectives of collective meanings which is a common characteristic in the Chinese culture^5^. Hong Kong Chinese were more willing to cooperate with friends^6^ and made external rather than internal causal attribution in an ambiguous situation^2,7^, after presented with a Chinese than Western culture related pictures. The pronouns ‘We’ and ‘I’could be used as the primes for collectivism and individualism behaviours, respectively^8,9^. These cultural priming results were also consistent with the situated culture model^10^ in which the activation of a specific culture depends on its situational relevance.

Both the dynamic multicultural view and the situated culture model emphasize the importance of recent cultural exposures in activating the culture-specific knowledge systems and predict spontaneous extractions and categorizations of culture-related information in the immediate environment. The current study examined spontaneous categorizations of cultural information in the environment by measuring an event-related brain potential (ERP) component called the mismatch negativity (MMN)^11,12^.

The human brains continuously monitor the surroundings for unexpected changes. Common properties or regularities shared among the typical or standard events are extracted for predicting future events^13^. Deviant events violating the regularities or expectations automatically elicit the MMN for signaling remedial actions. A generic fronto-sensory cortical network with forward and backward connections^14–17^ underlying a prediction violation mechanism in MMN generation across sensory modalities was suggested^18^. The MMN is not only sensitive to changes in physical features like colors^19^ or pitch of tones^20^, but also to changes in emotion^21^, abstract color properties^22^, pitch interval rules^23,24^, feature conjunction rules^25^, or social categories, such as gender^26,27^. Although, MMN to deviants in cultural category had not been demonstrated in the literature, MMN can be elicited by deviants violating an expected perceptual category^28^. As this deviant detection process does not require attentional efforts, MMNs are regarded as the intrinsic biomarker for automatic or spontaneous categorization.

In laboratory settings, a visual passive oddball paradigm^18,29^ is typically used to elicit MMNs in the visual modality (vMMN or visual mismatch responses, vMMR; from here, we adopt the nomenclature that MMN refers to the general, non-sensory specific mismatch brain response elicited in pre-attentive change detection, while aMMN refers to the MMN in auditory modality). Participants engage in a task presented at the foveal region of the visual field, while task-irrelevant infrequent deviants are randomly presented among a stream of frequent standards in the peripheral visual field. To dissociate MMNs from the sensory or early ERP components, the MMNs are typically measured from the difference waveforms calculated by subtracting the ERPs to the deviants with those to the standards^30^. The vMMN peaks from 100 to 400 ms after the onset of perceptual deviance (which is not necessarily aligned with the stimulus onset) with a central and/or right posterior scalp distribution^31^ depending on the deviance properties. Deviance in simple physical properties (e.g., color, orientation) typically elicited an early mismatch response (< 200 ms) with right posterior scalp distribution, while deviance in abstract properties (e.g., semantics) elicited vMMNs with a delayed latency (around 400 ms) and a central scalp distribution^18,32–34^(see^35,36^ for an alternative view). The differences of the vMMN latencies and scalp distributions in detecting abstract versus physical changes were related to increases in the complexity of cognitive processing and overall frontal cortex activities, as well as differences in the dynamics of the frontal-sensory cortical network^18,25,37^.

The MMN amplitude increases with degree of deviance^20,23,25^ and is modulated by psychological factors affecting the perceived categories. For example, individuals who are high in fluid intelligence are more sensitive to social information and showed larger vMMNs to change in emotional faces than individuals with average fluid intelligence^21^. The second aim of the current study is to examine individual differences in the sensitivity to cultural changes with MMN.

The cultural effects on cognition and behaviors were modulated by the belief about race^38^, which is also known as the racial essentialism. Racial essentialism is a subtype of psychological essentialism that is critical for understanding the underlying and identity-determining essences in social categorization^39^. Psychological essentialism also plays an important role in stereotyping and prejudice^39–41^. Compared to individuals with low psychological essentialism, individuals with high psychological essentialism are less flexible in social categorization, less likely to make situational dependent judgement, and more common to make internal attribution of others’ behaviours^42^.

Individuals who endorse or are high in racial essentialism believe that race is a fixed biological property or essence which determines a person’s characteristics and abilities. Low racial essentialism individuals believe that racial categories can be changed according to the economic or historical contexts. In other words, the characteristics and abilities of an individual are not confined by or attributed to his/her race, and can be altered. Racial essentialism affects racial categorization. Individuals with high racial essentialism were more sensitive to the facial skin tones associated with different races than those with low racial essentialism^43^. Racial essentialism is developed based on personal experiences (i.e., the lay theory of race^44^) and has impacts on the organizations of the culture-specific knowledge systems. Comparing to the low racial essentialism individuals, the high racial essentialism individuals had more distinct knowledge system for each culture and were more sensitive to cultural differences^38^. Based on this racial essentialism effect on the culture-specific knowledge systems, individual differences in the sensitivity to environmental cultural changes indicated by MMN would be expected.

**Figure 1:**
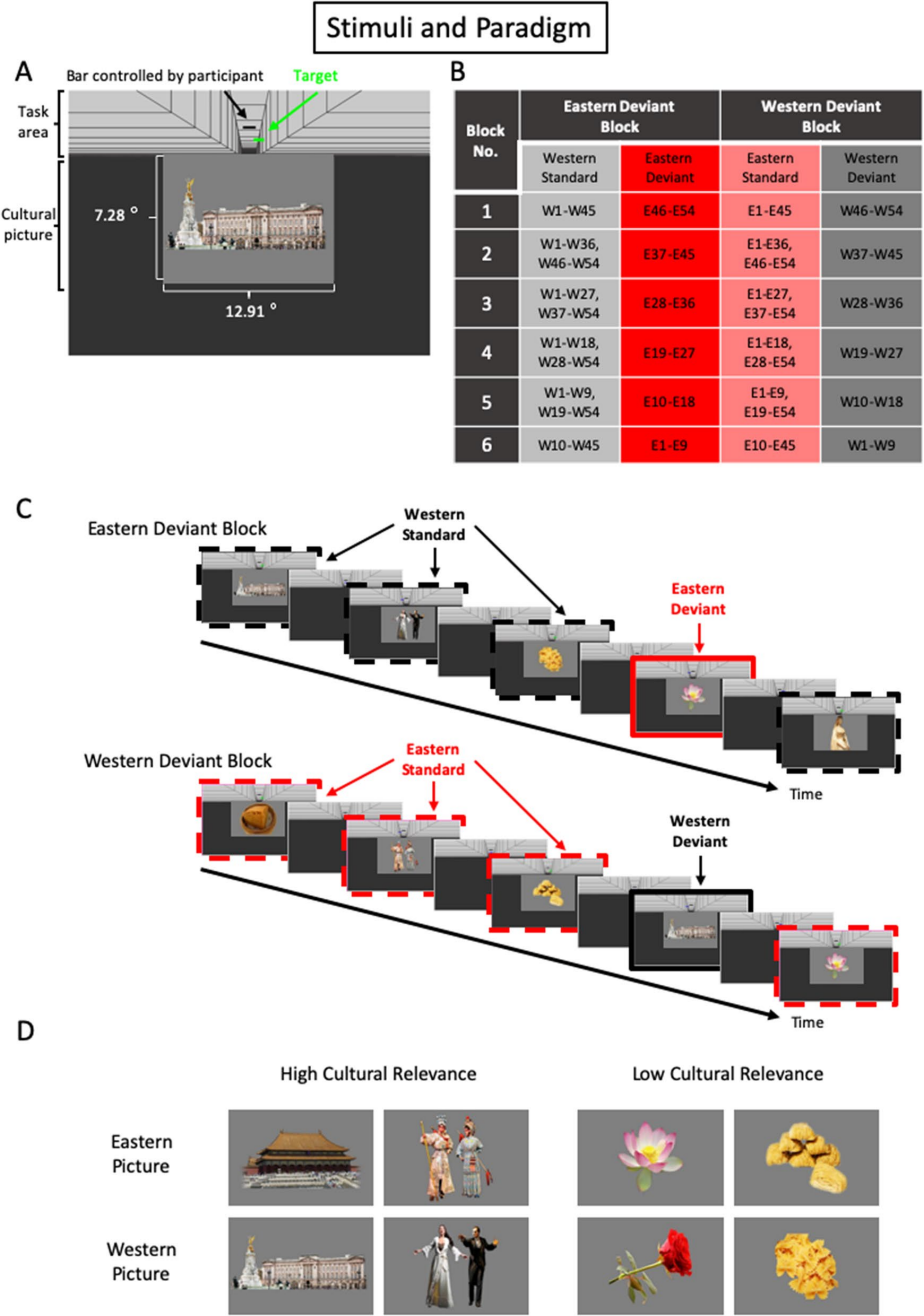
Stimulus properties and experimental design: (**A**) an illustration of the cultural picture (stimulus of the passive oddball paradigm) and the active video game task presented on the computer screen; (**B**) the organization of the 54 pairs of cultural pictures in the Eastern and Western deviant blocks (“E” indicates the Eastern pictures; “W” indicates the Western pictures; the number indicates a pair of pictures with the same theme); (**C**) schematic illustrations of the stimuli presented in the Eastern and Western deviant blocks; (**D**) examples of the Eastern and Western pictures with high or low cultural relevance.

The current study investigated: (1) whether cultural information in the environment could be spontaneously extracted and categorized, and (2) how racial essentialism would modulate spontaneous cultural categorization. Objects or scenes (symbols) with high or low relevance to the Eastern and Western cultures were presented in a passive visual oddball paradigm (Fig. 1). Deviant symbols with high Eastern (Western) cultural relevance presented in a stream of Western (Eastern) standard symbols are of larger deviance and were expected to elicit larger vMMNs compared to deviant symbols with low Eastern (Western) relevance. Individuals with high racial essentialism have more distinct knowledge system for each culture; they are expected to be more sensitive to cultural changes when compared to individuals with low racial essentialism. This study investigated the interaction of personal beliefs or attitudes and knowledge systems on spontaneous detections of environmental changes by the human brain.

## Results

### ERP results

**Figure 2:**
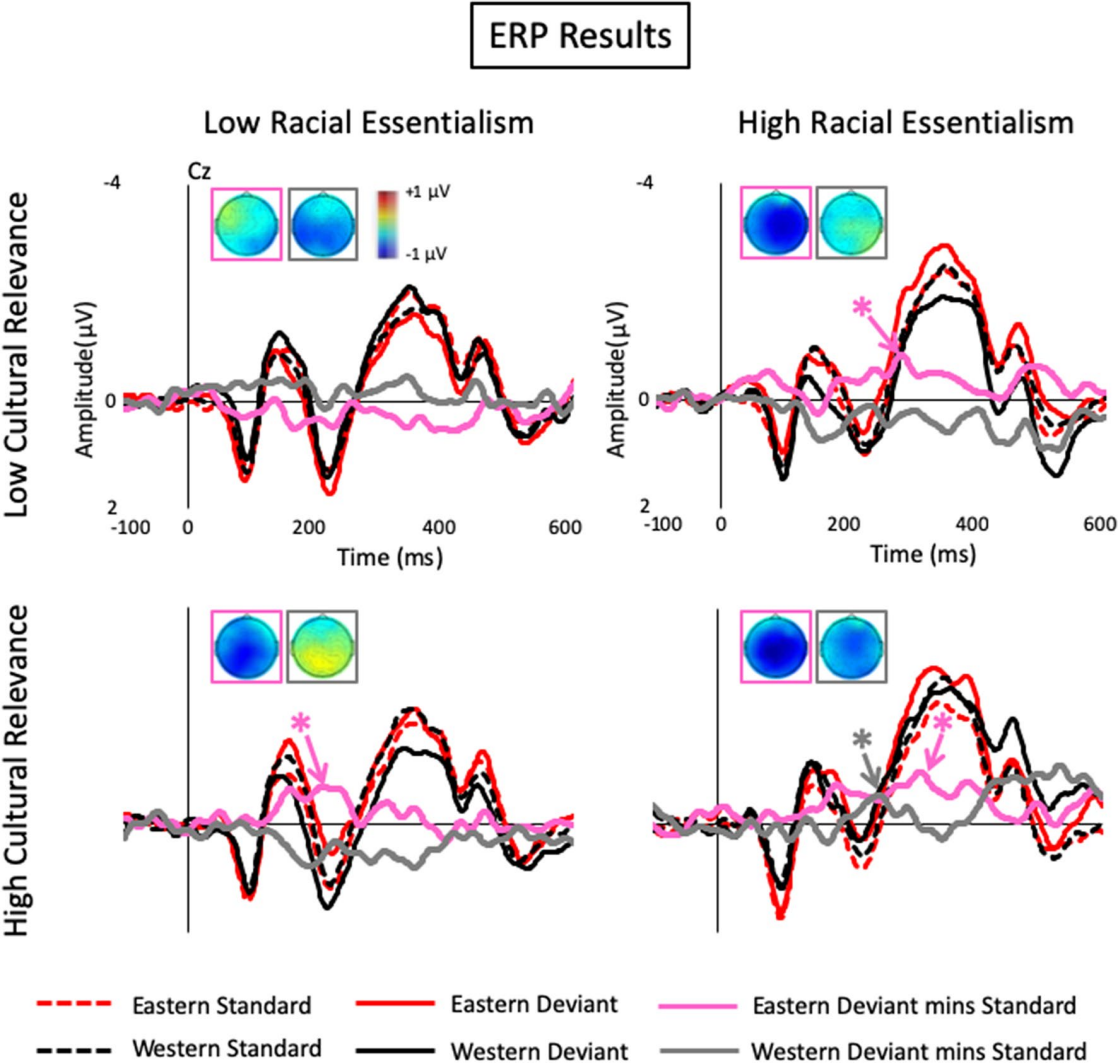
ERP waveforms to the standard and deviant cultural pictures and the deviant-minus-standard difference waveforms at the Cz electrode time-locked to the onset of stimuli. The scalp maps show a central distribution at the 40 ms time period of the peak vMMN responses. For individuals with high racial essentialism, vMMNs were elicited by both the Eastern and Western deviants with high cultural relevance, but only by the Eastern deviants with low cultural relevance. For individuals with low racial essentialism, vMMN was only elicited by the Eastern deviants with high cultural relevance. Positive ERP responses are plotted downward and represented by hot colors in the scalp maps. * indicates significant peak vMMN response (*p* < 0.05) in the 150 ms to 400 ms analysis time window.

**Figure 3:**
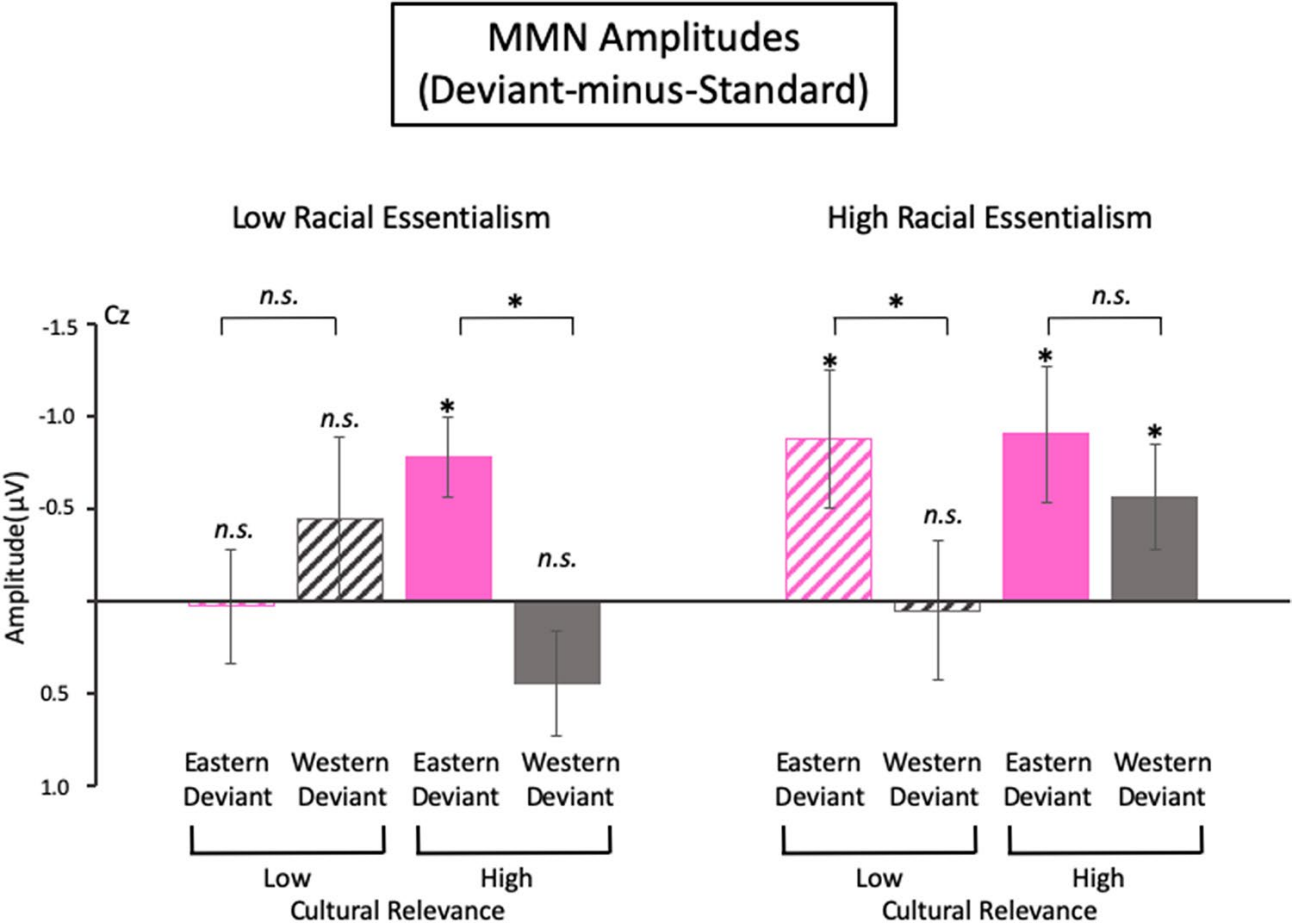
Amplitudes of the vMMN responses to deviants with high or low relevance to the Eastern and Western culture of the high or low racial essentialism individuals. Error bars indicate standard errors of the means. *p < 0.05; *n.s.,* not statistically significant.

Grand averaged ERP waveforms of the standard and deviant stimuli and their difference waveforms at the electrode showing the largest vMMN responses (i.e., the Cz electrode) were shown in Fig. 2. In general, vMMNs with a central scalp distribution were elicited in both racial essentialism groups, while the high racial essentialism group showed larger vMMNs to cultural changes than the low racial essentialism group (Fig. 3). Specifically, for the high racial essentialism group, the vMMNs elicited by the Eastern deviants with high cultural relevance (*t*(19) = − 2.37, *p* = 0.014, Cohenʼs d *(d)* = − 0.53 and the Eastern deviants with low cultural relevance (*t*(19) = − 2.47, *p* = 0.012, *d* = − 0.55) were statistically significant. The vMMN to the Western deviant with high cultural relevance (*t*(19) = − 1.98, *p* = 0.031, *d* = − 0.44) was significant, but not to the Western deviant with low cultural relevance (*t*(19) = 0.12, *p* = 0.55, *d* = 0.028). For the low racial essentialism group, vMMN was only observed to the Eastern deviant with high cultural relevance (*t*(19) = − 3.67, *p* < 0.001, *d* = − 0.82), but not to the Eastern deviant with high cultural relevance, or the Western deviants with high or low cultural relevance (*ts*(19) = − 1.02 to 1.55, *ps* = 0.16 to 0.48, *ds* = 0.018 to − 0.23). The effect sizes of the vMMNs ranged from − 0.44 to − 0.82, which were similar to the vMMNs that were calculated with properly controlled standards or control stimuli and elicited by abstract deviants^18,37,45^. A larger MMN effect size could be observed if the MMN was contaminated by the brain responses to the physical differences between the deviant and standard and/or the adaptation confound.

The mixed-effect ANOVA showed a main effect of culture type (*F*(1, 38) = 4.71, *p* = 0.036, η^2^_*p*_ = 0.11), while the main effects of cultural relevance and racial essentialism were not significant (*F*s(1,38) = 0.32 and 2.71, *p*s = 0.58 and 0.11, η^2^_*p*_s = 0.008 and 0.067). The two-way interaction effects between racial essentialism and culture type (*F*(1,38) = 0.32, *p* = 0.58, η^2^_*p*_ = 0.008), between racial essentialism and cultural relevance (*F*(1,38) = 0.54, *p* = 0.47, η^2^_*p*_ = 0.014), and between culture type and cultural relevance (*F*(1,38) = 1.33, *p* = 0.26, η^2^_*p*_ = 0.034) were not statistically significant. However, most importantly, a significant three-way interaction effect of cultural relevance, culture type, and racial essentialism (*F*(1,38) = 5.53, *p* = 0.024, η^2^_*p*_ = 0.13) was observed.

Follow-up repeated measure ANOVAs with the factors cultural relevance and culture type were carried out for the high and low racial essentialism groups, separately. In the low racial essentialism group, the main effects of culture type *(F*(1,19) = 1.09, *p* = 0.31, *η*^*2*^_*p*_ = 0.054) and cultural relevance (*F*(1,19) = 0.02, *p* = 0.89, *η*^*2*^_*p*_ = 0.001) were not statistically significant. However, the two-way interaction effect of cultural relevance and culture type was statistically significant (*F*(1,19) = 5.91, *p* = 0.024, *η*^*2*^_*p*_ = 0.24). Paired t-tests showed larger vMMN to the Eastern deviant with high cultural relevance than the Western deviant with high cultural relevance (*t*(19) = − 3.00, *p* < 0.001, *d* = − 0.67); while the vMMN difference between the Eastern and Western deviants with low cultural relevance was not significant (*t*(19) = 0.82, *p* = 0.42, *d* = 0.18). In the follow-up repeated measure ANOVA on the high racial essentialism group, the main effect of culture type was statistically significant (*F*(1,19) = 4.58, *p* = 0.045, *η*^*2*^_*p*_ = 0.19), indicating larger vMMNs evoked by the Eastern than the Western deviants. The main effect of cultural relevance (*F*(1,38) = 0.66,* p* = 0.43, *η*^*2*^_*p*_ = 0.034), as well as the interaction effect of cultural relevance and culture type (*F*(1,38) = 0.75, *p* = 0.40, *η*^*2*^_*p*_ = 0.038) were not significant. Paired sample t-tests showed larger vMMN to the Eastern deviant with low cultural relevance than the Western deviant with low cultural relevance (*t*(19) = − 2.27, *p* = 0.035, *d* = − 0.51), while the vMMN amplitude difference between the Eastern and Western deviants with high cultural relevance was not significant (*t*(19) = 0.71, *p* = 0.49, *d* = 0.16). Similar results and conclusions were obtained by the linear mixed-effect model analysis which is similar to the mixed-effect ANOVA except for treating racial essentialism as a continuous variable (Supplementary Analysis 1).

These vMMN results were not driven by the familiarity to the Western and Eastern cultural pictures; all the two-way interaction effects and the three-way interaction effect in a mixed-effect ANOVA on the vMMNs with the factors, racial essentialism, culture type, and familiarity level (high and low; based on the median score) were not statistically significant (*F*s(1,38) = 0.003 to 0.078 , *p*s = 0.78 to 0.96). Compared to the vMMNs, the positive mismatch responses (i.e., MMR) were less prominent and not modulated by the culture type, cultural relevance, and racial essentialism (Supplementary Analysis 2).

In summary, individuals with high racial essentialism were sensitive to the Eastern and the Western cultural deviants with high cultural relevance, but only to the Eastern deviants with low cultural relevance, while the individuals with low racial essentialism were only sensitive to the Eastern deviants with high cultural relevance. These vMMN results demonstrated top-down modulation of the spontaneous environmental change detection system by racial belief and cultural knowledge.

## Discussion

This study investigated (1) whether cultural information in the environment could be spontaneously extracted and categorized into the corresponding cultures, and (2) whether spontaneous cultural categorization would be modulated by individual differences in racial essentialism. Spontaneous categorization of cultural information was reflected by vMMN responses to cultural changes. The cultural categories of the standards and deviants were determined based on the culture-specific knowledge systems proposed by the dynamic multicultural view. From the prediction violation perspective of MMN^13^, the cultural category shared among the standards was used to predict the cultural category of future events. When a deviant violating the expected category was detected by the brain, MMN was automatically generated. Our results showed vMMNs to both Eastern and Western cultural deviants and a general increase in vMMN amplitude with larger deviation from the standard cultural category. These results demonstrated spontaneous extraction and categorization of information from the environment supporting the dynamic multicultural view.

In addition, for individuals with high racial essentialism, vMMNs were observed in detecting the Eastern deviants with high or low cultural relevance, and the Western deviants with high cultural relevance only. However, for individuals with low racial essentialism, vMMN was only found in detecting the Eastern deviants with high cultural relevance. The modulation of vMMN responses by racial essentialism demonstrated the influences of the racial belief on the spontaneous categorization of cultural information. Although the automatic change detection system reflected by MMN is designed to tackle changes in the external environment, this change detection system is subject to top-down modifications of cultural knowledge and racial beliefs.

Our results showed vMMNs to both Eastern and Western cultural deviants; however, the sensitivity to Eastern cultural change was higher than that to the Western cultural change. Although the participants experience both Eastern and Western cultures in daily life, it is reasonable to expect them having higher sensitivity to the Eastern culture which is more closely related to the dominant ethnic Chinese group than the Western culture in the local population. The sensitivity to cultural changes were further modulated by racial essentialism^46^. While individuals with low racial essentialism are less sensitive to cultural differences and were only able to detect the Eastern deviants, individuals with high racial essentialism are more sensitive to cultural differences and were able to detect both Eastern and Western deviants. The modulation of vMMN by the interaction of racial essentialism and culture types further our understanding on spontaneous categorizations of cultural information.

The lay theory of race^44^ suggested that the racial belief is developed from personal experiences. Based on this theory, the modulation of vMMN responses by racial essentialism is in line with previous studies showing modulation of the MMN sensitivity to changes by individual differences in experience^22,31,47^. For example, 6-month-old infants showed aMMNs to changes in phonemes that exist in both their native language and a foreign language. However, at 1 year old, aMMNs were only found in detecting changes of phoneme in the native language but not in the foreign language^48^. In another vMMN study, participants who are native in the language with specific names for colors that have small differences showed better color discrimination^22^. These results demonstrated the effect of language experiences on the sensitivities to changes. Similarly, a vMMN study showed that right-handed individuals were more sensitive to unexpected right-hand images than left-hand images^31^. Due to differences in personal experience, the standards and the deviants carried different meanings to an individual leading to differences in MMN responses. From the predictive coding perspective^13^, personal experiences may affect the weights or the importance of a prediction error leading to differences in prediction model updating and future predictions.

Racial essentialism is one of the psychological essentialisms^39^ in which a person’s attribute (e.g., intelligence and race) is believed to be a fixed and inheritable biological feature or essence. For example, individuals with high intelligence essentialism were more likely to make attribution to trait characteristic (e.g., I failed the test because I am dumb), while individuals with low intelligence essentialism make non-trait attribution (e.g., I failed the test because of my effort or strategy^42^). Individuals with high racial essentialism were more likely to categorize a person by race than other attributes, like occupations^46^. The current study demonstrated the possibility in applying vMMN to study the influence of psychological essentialism on social categorization.

Racial essentialism can be regarded as a kind of attitude or belief affecting the cultural information processing of the cognitive system. Attitude acts as a type of processing or cognitive styles affecting how the system handles information in general. Individual attitude differences have been reflected by explicit and implicit tasks, using self-reporting methods^49,50^, priming^49^, or implicit associative tests^50^. The results of the current study suggested top-down influence of attitude, or specifically the racial essentialism, on the sensitivities of the pre-attentive change detection system to cultural information changes. As reflected by the automatically generated MMN responses, attitudes could exert influences on cognitive processes in anticipating the incoming events at the perceptual stage, and not limited to the later decision making or response execution stages suggested by previous studies^38^.

Previous vMMN studies demonstrated spontaneous categorization of social information, such as emotion^21^, trustworthiness^51^, gender^26,27^, self versus others^34^. As the social information in these studies was carried by human faces or bodies, this categorization process involves extracting configural features from a single type of object. However, the categorization of cultural symbols in the current study requires extracting the common theme shared among different objects or scenes by applying the culture-specific knowledge. This is the first study to demonstrate vMMNs to cultural deviants indicating spontaneous categorization of cultural symbols based on elaborated cultural knowledge systems.

Spontaneous categorizations of cultural symbols were indirectly demonstrated by using priming procedure in previous studies^2,5^. Typically, task-irrelevant cultural symbols were presented as the primers and followed by a cognitive task. The impact of culture on cognition were reflected by the task performance which is the final outcomes from a series of cognitive processes, including sensory registration, recognition, evaluation, and decision making; while the spontaneous categorizations of cultural information at a certain stage was inferred through this priming procedure. The current study is different from previous studies to reveal automatic processing of cultural information at the pre-attentive perceptual stage by using the vMMN.

P3 responses and differences between the deviants and standards could be observed in the passive oddball paradigms of MMN studies when the active task cannot fully capture the attention of participants. However, as mentioned in the methods session, none of the participants reported detecting any order or pattern in presenting the cultural pictures at the end of the experiment, the passive paradigm manipulation seems to work as expected. In addition, the examinations of the ERP waveforms, the ERP response pattern, and the statistical analysis showed that the mismatch responses in the results cannot be explained by frontal P3 differences in the deviance detection process (see Supplementary Analysis 3).

## Conclusions

The current study demonstrated spontaneous categorizations of cultural information and modulations of this categorization process by racial essentialism with vMMN. Our findings provided neural evidence for the culture-specific knowledge systems proposed by the dynamic multicultural view and enhanced our understanding of psychological essentialism in social categorization and culture-specific knowledge. The interaction of spontaneous cultural categorization and the belief of racial origin revealed the holistic nature of the conscious and unconscious minds.

## Methods

### Participants

Forty university students (18 females, aged 18–26, mean age of 21.13 years), who are Chinese, lived and educated in Hong Kong since birth, participated after giving informed consents. This study was approved by The Joint Chinese University of Hong Kong—New Territories East Cluster Clinical Research Ethics Committee and performed in accordance with the relevant guidelines/regulations and the Declaration of Helsinki. All participants were right-handed according to the Edinburgh Handedness Inventory^52^, had either normal or corrected-to-normal vision, and no history of neurological disease.

### Racial essentialism

Racial essentialism was measured from the participants using the Lay Theory of Race Scale^44^. This instrument consists of eight items (e.g., “To a large extent, a person’s race biologically determines his or her abilities and traits”). Each item is measured with a 6-point Likert scale (1 = strongly disagree, 6 = strongly agree). An averaged racial essentialism score of 3.5 is regarded as the neutral point in the scale. Half of the items are reversely scored (e.g., “Races are just arbitrary categories and can be changed if necessary”). Higher average item scores indicate stronger endorsement of the biological racial origin or racial essentialism. Based on the median of the racial essentialism scores, participants were divided into the high and low racial essentialism groups (20 per group; high group: mean score = 4.13, SD = 0.55; low group: mean score = 2.69, SD = 0.42; *t*(38) = 9.35, *p* < 0.001, Cohenʼs d *(d)* = 2.96). The *t*-test against 3.5 results showed that the mean racial essentialism scores of the high and low groups were statistically different from the neutral opinion score (high group: *t*(19) = 5.12, *p* < 0.001, *d* = 1.15; low group: *t*(19) = − 8.73, *p* < . 001, *d* = − 1.95). The high racial essentialism group agreed that race is a biological essence while the low racial essentialism group disagreed with this biological determinism hypothesis of race.

### Stimuli and experimental design

During the experiment, participants played a video game presented at the upper part of the computer screen and ignored cultural pictures that were irrelevant to the video game presented in the lower part of the screen^29^. In the video game (Fig. 1A), blue or green rectangles (50% chance for each color) randomly appeared at the left, middle, or right positions of the perceptually farthest end of the canyon (i.e., near to the center of the screen) and moved to the perceptually closer end of the canyon (i.e., the top part of the screen), then disappeared. The task for the participants was to move a black rectangle located at the perceptually closer end of the canyon horizontally using the left and right arrow keys to catch the green rectangles and avoid the blue rectangles. For optimal performance, participants would fixate at the farthest end of the canyon where the blue or green rectangles first appeared. Both racial essentialism groups performed with higher than 90% accuracy in the video game task and showed no difference in their performance (*t*(38) = 0.54, *p* = 0.59, *d* = 0.17).

**Figure 4:**
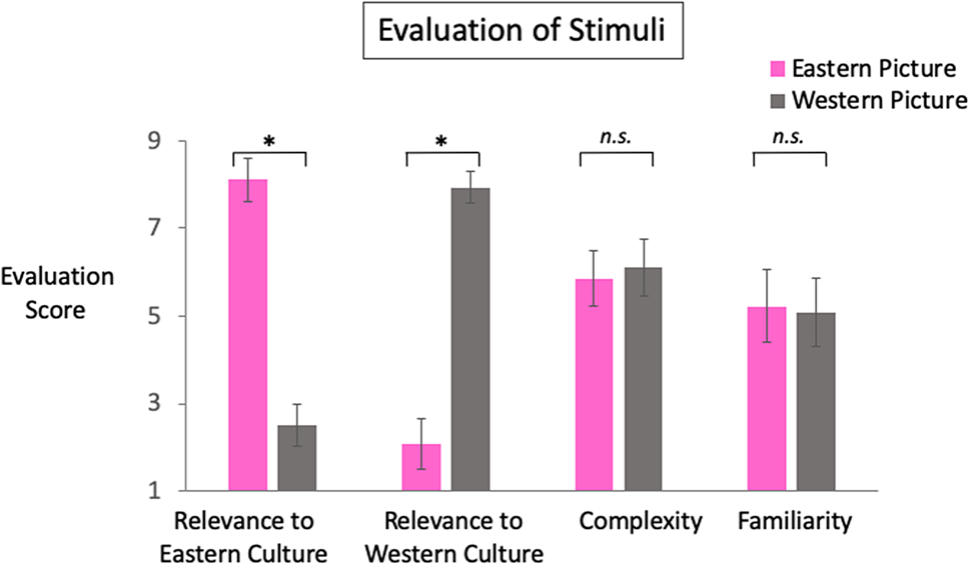
Evaluation results of the Eastern and Western picture stimuli. The Eastern picture stimuli showed higher relevance to the Eastern culture than that of the Western culture, while the Western picture stimuli showed higher relevance to the Western culture than that of the Eastern culture. The Eastern and Western pictures were not different in their complexity and familiarity ratings. *p < 0.05; *n.s.,* not statistically significant.

Fifty-four pairs of Eastern and Western cultural pictures (labelled as E1 to E54 and W1 to W54, respectively; Fig. 1B) were presented in a passive visual oddball paradigm at the lower part of the screen for eliciting the cultural vMMN. Each pair of the Eastern and Western pictures (e.g., E1 and W1) shared the same theme (e.g., wedding dress, palace, musical instrument). Sixty-eight university students (38 females, aged 18–23, mean age of 20.05 years) evaluated each picture for the relevance to Eastern culture (1 = very irrelevant; 9 = very relevant), relevance to Western culture (1 = very irrelevant; 9 = very relevant), complexity (1 = very simple; 9 = very complex), and familiarity (1 = very unfamiliar; 9 = very familiar) with a 9-point Likert scale. The Eastern pictures were rated significantly higher than 5 in the relevance to Eastern culture (mean score = 8.11, SD = 0.49; *t*(53) = 46.67, *p* < 0.001, *d* = 6.35; Fig. 4) and lower than 5 in the relevance to Western culture (mean score = 2.51, SD = 0.48; *t*(53) = − 37.78, *p* < 0.001, *d* = − 5.19); while the Western pictures were rated significantly higher than 5 in the relevance to Western culture (mean score = 7.94, SD = 0.37; *t*(53) = 57.84, *p* < 0.001, *d* = 7.95) and lower than 5 in the relevance to Eastern culture (mean score = 2.08, SD = 0.58; *t*(53) = − 37.96, *p* < 0.001, *d* = − 5.12). A differential cultural relevance score for the Western (Eastern) pictures was calculated by subtracting the relevance to the Eastern (Western) culture score from the relevance to the Western (Eastern) culture score. Based on the median of this differential score, the pictures were divided into the high and low cultural relevance groups. The differential cultural relevance scores were significantly higher for the high than the low cultural relevant Eastern or Western pictures (mean scores (SD) of the high and low cultural relevant Eastern pictures = 6.70 (0.21) and 5.36 (1.07), respectively, *t*(52) = 6.24, *p* < 0.001, *d* = 1.74; mean scores (SD) of the high and low cultural relevant Western pictures = 6.09 (0.28) and 4.77 (0.60), respectively, *t*(52) = 10.17, *p* < 0.001, *d* = 2.82), while their differences between the Eastern and Western pictures were not statistically significant (*F*(1, 104) = 0.005, *p* = 0.95, *η*^*2*^_*p*_ = 0.00). In addition, the Eastern and Western pictures were not different in the complexity and familiarity ratings (*t*s(106) = − 1.75 and − 0.14, *p*s = 0.08 and 0.89, *d*s = − 0.37 and − 0.17; Fig. 4).

The physical features, the intensity/luminance, the overall color measured by the proportional contribution of the Red, Green, and Blue (RGB) channels, the number of proto-objects which indicates the level of visual clutter, and the mean information gain^53^ which reflects the two-dimensional complexity, were calculated for each picture. Item level ANOVAs with the factors cultural relevance (high and low) and culture type (Eastern and Western) showed non-significant main effects or interaction effects on the intensity (*F*s(1, 104) = 0.41 to 2.62, *p*s = 0.11 to 0.53, η^2^_*p*_s = 0.004 to 0.025), the overall color (*F*s(1, 104) = 1.08 to 0.075, *p*s = 0.30 to 0.79, η^2^_*p*_s = 0.010 to 0.001), the number of proto-objects (*F*s(1, 104) = 0.29 to 2.31, *p*s = 0.13 to 0.59, η^2^_*p*_s = 0.003 to 0.022), and the mean information gain (*F*s(1, 104) = 3.31 to 0.15, *p*s = 0.072 to 0.70, η^2^_*p*_s = 0.031 to 0.001), suggesting comparable physical features between the Eastern and Western pictures with high or low cultural relevance.

The cultural pictures were presented in the Eastern and Western deviant blocks. In the Eastern deviant block, the Eastern cultural pictures served as the deviants while the Western pictures served as the standards, and vice versa in the Western deviant block (Fig. 1C). The 54 pairs of cultural pictures were divided into 6 subsets, with 9 pairs of pictures in each subset. In each deviant block, five subsets were presented as the standards while the remaining subset served as the deviants. There were 6 unique versions of the Eastern or Western deviant blocks; each version had a different subset of cultural pictures as the deviants. The themes of the deviants were matched between an Eastern and Western deviant block pair. The Eastern (Western) cultural vMMNs were measured from the difference waveforms calculated by subtracting the ERP waveforms to the Eastern (Western) standards presented in the Western (Eastern) deviant blocks from that to the Eastern (Western) deviants presented in the Eastern (Western) deviant blocks. The standards and deviants used for calculating the vMMNs were physically identical and presented with the same probability to prevent contamination of the vMMNs by brain responses to differences in physical features or the sensory adaptation confound in traditional oddball paradigm design^23,54^.

The cultural pictures were presented on the lower half of the screen with a dark-gray color background and a viewing distance of 110 cm. The size of the picture spanned 12.91 degree of visual angle horizontally and 7.28 degree vertically. The cultural pictures were presented for a duration of 310 ms followed by a blank screen of 510 ms; the stimulus onset asynchrony (SOA) was 820 ms. The 54 pictures of each block were repeated 5 times to produce a total of 270 trials in each version of the Eastern/Western deviant block, including 45 deviants (17% of trials) and 225 standards (83% of trials). Each version of the deviant blocks was presented to each participant once producing a total of 540 deviant and 2700 standard trials in the entire experiment (i.e., 135 deviant trials for each of the Eastern or Western deviant with high or low cultural relevance; Fig. 1D). There were 102.35 to 96.70 trials (averaged across participants) for each of the trial type in the analyses (Table 1). The trial number for each trial type in the analyses varied from participant to participant. To ensure that the results were not affected by the variation in trial numbers, analyses on the vMMNs by resampling the minimum trial number for each trial type across all participants (i.e., 52 trials) from the trials of each participant were carried out. The results of the resampling analyses were similar to the results presented above and the conclusions were identical.

**Table 1:** Numbers of trials in the ERP analyses.

Racial essentialism	Low				High			
Picture type	Eastern		Western		Eastern		Western	
Cultural relevance	Low	High	Low	High	Low	High	Low	High
Mean	100.15	100.65	100.00	99.55	102.35	96.70	99.70	100.60
Standard Deviation	21.30	19.17	20.39	20.08	17.10	17.16	18.67	18.68
Minimum	64	61	59	52	71	64	68	64
Maximum	128	126	130	130	125	121	126	128

The pictures were presented in pseudorandom order with the constraints that the deviants were preceded by two or more standards. All of the visual stimuli were presented on a 24-inch LCD monitor with 100 Hz refresh rate. The presentations of the stimuli in the video game were adjusted to avoid synchronization with the onset of the cultural pictures. The Eastern and Western deviant blocks were presented alternatively, while their presentation order was counterbalanced across participants. The presentation order of the 6 versions of each block type was randomized for each participant. At the end of the experiment, none of the participants reported detecting any order or pattern in presenting the cultural pictures.

### ERP recording and analyses

The Electroencephalogram (EEG) was recorded with Ag/AgCl electrodes (EasyCap, Herrsching, Germany) at 64 locations according to the modified 10–20 system using the EGI 200 amplifier (Electrical Geodesics Inc., USA). Three additional electrodes, two placed on the left and right outer canthi and one below the right eye were used for horizontal and vertical electrooculargram recordings, respectively. The left mastoid and AFz electrodes served as the online reference and the ground electrode, respectively. The impedance of all electrodes was kept below 10 kOhms. The EEG was recorded with a sampling rate of 500 Hz, and online band-pass filtered at 0.01–200 Hz. EEG was re-referenced to the nose electrode and offline band-pass filtered at 0.01–30 Hz with a 6 dB roll off. Subsequent data analyses were carried out using the EEGLAB^55^ and ERPLAB^56^ toolboxes of the MATLAB (MathWorks, Natick, MA).

The EEG waveforms were segmented into epochs of 1000 ms with a 400 ms pre-stimulus onset period. Epochs with ocular artifacts in the 400 ms pre-stimulus onset to 500 ms post-stimulus onset period were rejected from the analysis. The mean voltage during the 100 ms pre-stimulus interval was used as the baseline. Epochs with a voltage change larger than 100 μV on any channel were excluded from further analysis. The ERPs of the standard and deviant for each trial type were averaged separately for each participant.

To capture the complex MMN response generated from a fronto-sensory cortical network^14,17,18,20,57^, a difference waveform approach^58^ is commonly used to dissociate the MMN from the sensory or early ERP components. Similar to most MMN studies, difference waveforms were calculated by subtracting the ERPs of deviants with that of the physically identical standards for each condition in the current study. Based on the procedure in previous studies^18,59,60^, vMMNs were measured as an averaged response across a 40 ms period from the difference waveform of each condition. The 40 ms averaging period was centered at the peak negative response in a 150 to 400 ms time window after the stimulus onset. Statistical analyses were carried out at the electrode showing the largest vMMN responses (i.e., the Cz electrode). Previous studies also demonstrated largest vMMN responses to self-related information^34^, motion^32^, and facial expression^33^ changes at the Cz electrode. Consistent with the MMN review in the introduction, vMMNs with a central scalp distribution is commonly found in detecting abstract deviance. Cultural categories are regarded as a type of abstract properties and vMMNs to cultural changes were expected to have a central distribution. The vMMNs at the Cz electrode were representative of the vMMN response pattern observed in this study. The central vMMN scalp distribution was also supported by the vMMN analyses on the right posterior electrodes (see Supplementary Analysis 4).

The latency of 150 to 200 ms from the onset of the stimuli^58^ was commonly used for measuring aMMNs to deviants that were physically different from the standards (e.g., frequency/pitch or loudness of tones). However, studies on the MMN and perceptual differences^61^ showed that the MMN latency should be aligned to the onsets of perceptual differences between the deviants and the standards. The latency of MMN to semantic category change can be up to 400 ms after the visual stimulus onset^62^. Our recent study^18^ and others^63^ also showed a similar delay in vMMN latency to an abstract property deviance (around 400 ms after stimulus onset) compared to a physical property deviance (around 200 ms after stimulus onset). A similar delay in MMN latency would be expected in detecting cultural category change in the current study. As this is the first study investigating the vMMNs to cultural changes and the exact time window for vMMN measurement was unknown, a peak response measurement approach with a 150 to 400 ms time window was used.

One-sample t-tests (one-tailed) against zero were used to test the presence of vMMNs. To compare the vMMNs elicited in different conditions, a mixed-effect analysis of variance (ANOVA) with two within-subject factors, cultural relevance (high and low), culture type (Eastern and Western), and one between-subject factor, racial essentialism (high and low group) was conducted. Follow-up ANOVAs and t-tests on the vMMN amplitudes were carried out.

To examine whether the results of the current study is produced by the peak amplitude measurement procedure, the vMMNs were analyzed in non-parametric randomization tests. The non-parametric randomization test results were similar to the original mixed-effect ANOVA results (Supplementary Analysis 5).

## Supplementary Information

Supplementary Information.
