# Supplementary material for: Belief in biological origin of race (racial essentialism) increases sensitivities to cultural category changes measured by ERP mismatch negativity (MMN)
Source: Sci Rep. 2022 Mar 15;12:4400. doi: 10.1038/s41598-022-08399-3 (PMC8924166; doi:10.1038/s41598-022-08399-3)
Supplement: Supplementary file 1 — Supplementary Information. [file 41598_2022_8399_MOESM1_ESM.pdf]

Supplementary Material

Belief in Biological Origin of Race (Racial Essentialism) Increases Sensitivities to Cultural Category Changes Measured by ERP Mismatch Negativity (MMN)

[1,2#]Ginger Qinghong ZENG, [2,3#]Xue-Zhen XIAO,[4]Yang WANG, and [4]Chun-Yu TSE

[1] Institute of Advanced Technology,
University of Science and Technology of China

[2]Department of Psychology,
The Chinese University of Hong Kong, Hong Kong

[3] School of humanities and social science,
The Chinese University of Hong Kong, Shenzhen

[4] Department of Social and Behavioral Sciences,
City University of Hong Kong, Hong Kong SAR, China

# These authors contributed equally.

Address all correspondence to:

Chun-Yu TSE
Assistant Professor
Department of Social and Behavioral Sciences
City University of Hong Kong
Address: YEUNG-Y7516, City University of Hong Kong, Kowloon, Hong Kong SAR, China
Email: chunyu.tse@cityu.edu.hk
Tel: (852) 3442 4533
Fax: (852) 3442 0283

Supplementary Analysis

1. Linear Mixed-effect Model Analysis

The linear mixed effect model was used to analyze the effects of racial essentialism, as a continuous variable, while both cultural relevance (high cultural relevance coded as +1 and low cultural relevance coded as -1) and culture type (Eastern culture coded as +1; Western culture coded as -1) as categorical variable, and their interaction effects. Specially, the full model, vMMN ~ Culture Relevance * Culture Type * Racial Essentialism + (1 |subject), was estimated and reported below. Identical result was obtained with the model vMMN ~ Culture Relevance * Culture Type * Racial Essentialism + (Racial Essentialism |subject) when racial essentialism was estimated as a random effect. The main effects of racial essentialism (*beta* = -0.21, $t(152) = -0.78$, $p = .44$), cultural relevance (*beta* = -1.19, $t(152) = -0.88$, $p = .38$), and culture type (*beta* = -1.97, $t(152) = -1.45$, $p = .15$) on vMMNs were not statistically significant. The two-way interaction effects between racial essentialism and cultural relevance (*beta* = 0.23, $t(152) = 0.59$, $p = .56$), and between racial essentialism and culture type (*beta* = 0.64, $t(152) = 1.67$, $p = .097$) were not statistically significant. However, the two-way interaction effect of cultural relevance and culture type (*beta* = 4.59, $t(152) = 2.40$, $p = .018$), and the three-way interaction effect of racial essentialism, cultural relevance, and culture type (*beta* = -1.18, $t(152) = -2.17$, $p = .031$) were statistically significant. The vMMN response pattern and the results from the linear mixed effect model analysis were similar to that of the mixed-effect ANOVA shown in Figure 3.

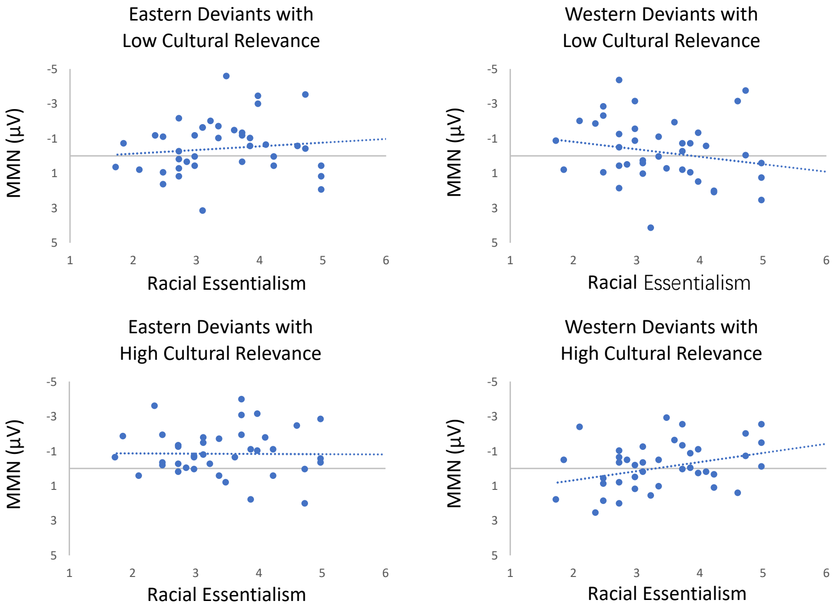

Supplementary Figure 1. The vMMN response pattern of the linear mixed effect model analysis was similar to that of the mixed-effect ANOVA shown in Figure 3.

2. Positive Mismatch Response / MMR

Most vMMN studies reported negative mismatch responses[1], but some studies reported positive mismatch responses[2,3] (MMR). Since no prior study examined the vMMN to cultural changes, the positive responses at the Cz electrode in the vMMN time window were analyzed with procedures similar to that in analyzing the negative mismatch responses, except for measuring the positive instead of the negative response. One-sample *t*-tests showed significant positive mismatch responses to Western deviants with high cultural relevance in the low racial essentialism individuals ($t(19) = 2.11$, $p = .024$, $d = 0.50$) and Western deviants with low cultural relevance in the high racial essentialism

individuals ($t(19) = 1.92$, $p= .036$, $d = 0.43$), while no positive mismatch responses were found in other conditions (ts(19) = -1.04 to 1.68, ps= .11 to .16, ds = -0.23 to 0.38). The mixed-effect ANOVA with the factors culture type, cultural relevance, and racial essentialism showed that all the main effects, two-way and three-way interaction effects were not statistically significant (Fs(1,38) = 0.078 to 2.38 , ps = .13 to .78). Compared to the negative mismatch responses, the positive mismatch responses were less prominent and not modulated by the culture type, cultural relevance, and racial essentialism.

Assuming that the automatic detection system is less sensitive to the deviants with low cultural relevance and the Western deviants, as the participants were more used to the Eastern culture, the positive mismatch response elicited by the Western deviant with low cultural relevance could indicate an ambiguous or more difficult to-be-detected deviant. This proposal is consistent with the positive mismatch response observed in the Western deviant with high cultural relevance in low racial essentialism individuals. Previous studies repeatedly demonstrated differences in the spatiotemporal dynamics of the fronto-sensory cortical network in detecting deviants with various degree of deviance[4–7]. The detection of ambiguous deviants typically involved additional early frontal mismatch responses than detecting salient deviants[5,7,8]. The association of the mismatch response polarities and sensitivities of the brain in detecting cultural changes needs to be clarified in future studies.

3. Frontal P3 as Alternative Explanation

At the Cz electrode (Figure 2; positive response is plotted downward), the positive responses from 200 ms to 270 ms of the ERP waveforms (non-subtracted waveforms) were

too early to be the frontal P3. The time window of typical frontal P3 was dominated by negative responses from 280 ms to 500 ms. Similarly, P3 component was absent at the Fz electrode in all the conditions (Supplementary Figure 2).

Consistent with typical MMN findings, our results showed increases in negative responses to the deviants when compared to the standards at the Cz electrode. This increase in negative response pattern was different from the typical P3 response observed in active oddball paradigm. Increases in P3 amplitude (i.e., increases in a positive response) to deviants are typically observed when compared to the standards.

At last, mixed-effect ANOVA with two within-subject factors, cultural relevance (high and low), culture type (Eastern and Western), and one between-subject factor, racial essentialism (high and low group) were carried out on the differences in the P3 responses between the deviants and standards at the Fz electrode. Similar to the MMN analyses, the difference in P3 responses was a mean amplitude measure across a 40 ms period centered on the positive peaks in the P3 time windows (300 ms to 500 ms) in the difference waveforms. All the main effects and interaction effects at the Fz electrodes were not statistically significant (Fs(1,38) = 0.05 to 3.66, ps = .06 and .83, η^2_ps = .001 to .088).

Due to the absence of frontal P3 in the ERP waveforms, responses incompatible with frontal P3, and the absence of statistical evidence supporting modulation of frontal P3, the ERP responses observed in the current study cannot be explained as frontal P3 differences.

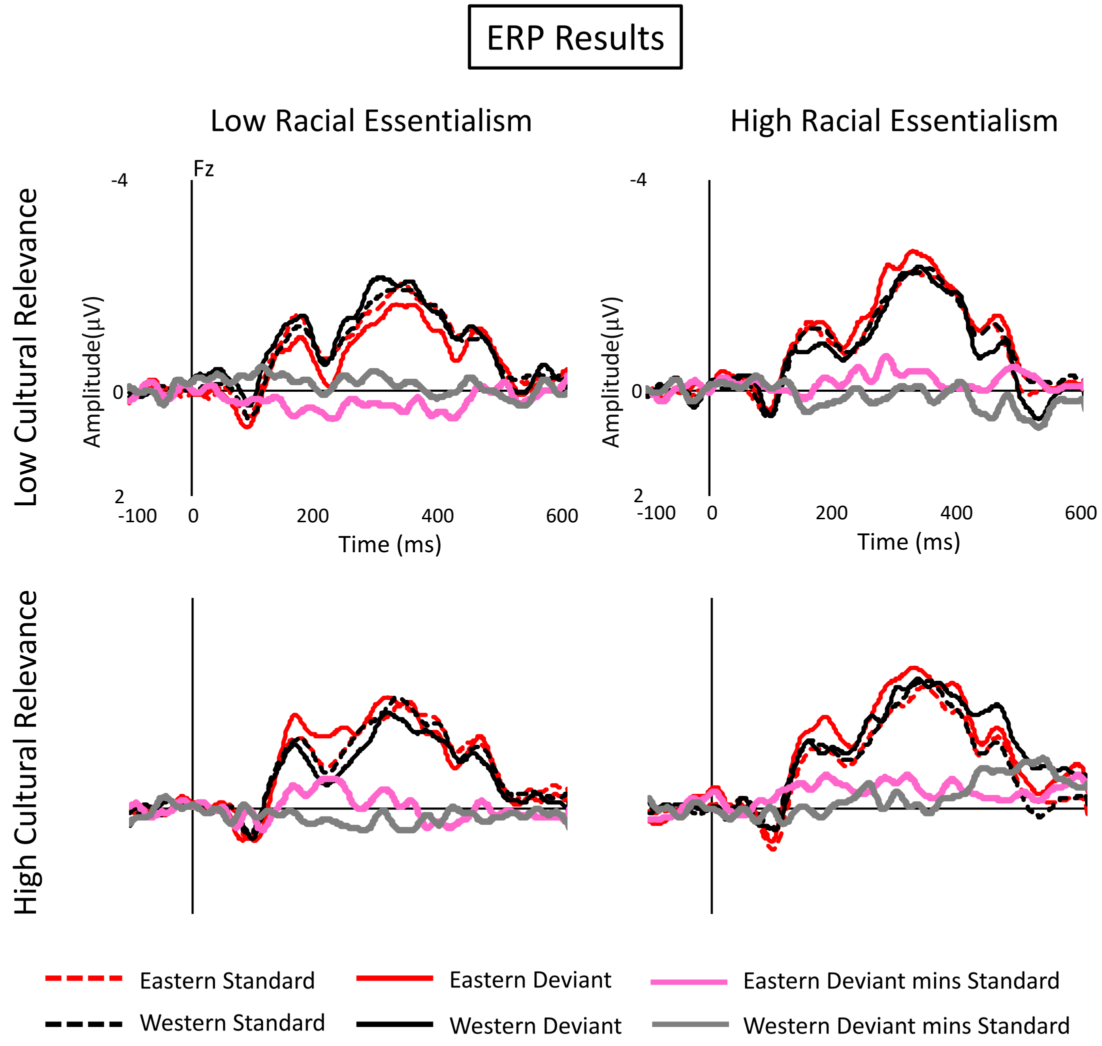

Supplementary Figure 2. ERP waveforms to the standard and deviant cultural pictures at the Fz electrode time-locked to the onset of stimuli. Positive ERP responses are plotted downward. Consistent with the ERP responses at the Cz electrode shown in Figure 2, frontal P3 was absent in all conditions.

4. Support for Analyses of the Cz vMMNs

The vMMNs at the Cz electrode were representative of the central vMMN responses observed in this study. The vMMN patterns and results of the mixed-effect ANOVA (with the factors culture type, cultural relevance, and racial essentialism) carried out on the vMMNs averaged across the 6 frontocentral electrodes (FC1, FC2, FCz, C1, C2, Cz) or 14 electrodes (F1, F2, F4, F6, Fz, FC1, FC2, FC3, FC4, FC6, FCz, C1, C2, Cz) showing the largest vMMN responses were similar to that of the Cz electrode (see 4.1 and 4.2 for details). These results suggested that the vMMN response pattern at the Cz electrode can reliably represent the overall vMMN responses observed in the current study. The reliability of the Cz vMMN results was further demonstrated by the results of the non-parametric randomization tests (See Supplementary Analysis 5 below).

The central vMMN scalp distribution observed in the current study was also supported by the absence of vMMN at the right posterior electrodes, specifically the PO8 and O2 which demonstrated vMMN responses in previous studies[9,10] . One-sample t-tests against zero showed a statistically significant overall vMMN response (averaged across all conditions and participants) at the Cz electrode ($t(39) = -8.35, p < .001, d = -1.32$), while such an overall vMMN response was not found at the PO8 ($t(39) = 4.08, p = .99, d = 0.65$) or O2 electrode ($t(39) = 2.87, p = .99, d = 0.45$). In addition, all the main effects, the two-way and three-way interaction effects of the two mixed-effect ANOVAs (with the factors culture type, cultural relevance, and racial essentialism) were not statistically significant for the vMMN responses at the PO8 (Fs(1,38) = 0.002 to 3.50, ps = .069 to .97, η^2_ps= <.001 to .084) and O2 (Fs(1,38) = 0.35 to 2.18, ps = .15 to .85, η^2_ps= .001 to .054) electrodes.

4.1. 6 Electrodes vMMN Results

For the vMMNs averaged across the 6 frontocentral electrodes, the mixed-effect ANOVA showed a significant main effect of culture type (F(1, 38) = 4.94, p = .032, η^2_p = .12), while the main effects of cultural relevance and racial essentialism were not significant (Fs(1,38) = 0.43 and 3.35, ps = .52 and .05, η^2_ps = .011 and .081). The two-way interaction effects between racial essentialism and culture type (F(1,38) = 0.46, p = .50, η^2_p = .012), racial essentialism and cultural relevance (F(1,38) = 0.21, p = .65, η^2_p = .006), and between culture type and cultural relevance (F(1,38) = 1.24, p = .27, η^2_p = .032) were not statistically significant. However, most importantly, a significant three-way interaction effect of cultural relevance, culture type, and racial essentialism (F(1,38) = 4.43, p =.042, η^2_p = .10) was observed.

Follow-up repeated measure ANOVAs with the factors cultural relevance and culture type were carried out on the vMMNs for the high and low racial essentialism groups, separately. For the low racial essentialism group, the main effects of culture type and culture relevance were not significant (Fs(1,38) = 1.17 and 0.02, ps= .29 and .88, η^2_ps = .06 and .001), while the two-way interaction effect between culture type and cultural relevance was significant (F(1,38) = 4.43, p = .049, η^2_p = .19). There was no significant difference between the vMMNs to the Eastern and Western deviants with low culture relevance (t(19) = 0.87, p = .39 , d = 0.20). However, the vMMNs to the Eastern deviants with high culture relevance was larger than that of the Western deviants with high culture relevance (t(19) = -3.01, p = .007, d = -0.67).

For the high racial essentialism group, only the main effect of culture type was marginally significant (F(1,38) = 4.31, p = .052, η^2_p = .19). The main effect of culture

relevance and the two-way interaction between culture type and cultural relevance were not significant (Fs(1,38) = 0.51 and 0.59, ps = .48 and .45, η^2_ps = .026 and .030). For the low culture relevance, there was a larger vMMNs to the Eastern than the Western deviants (t(19) = -2.13, p = .047, d = -0.48). For the high culture relevance, no significant difference between the vMMNs to the Eastern than the Western deviants (t(19) = -0.72, p = .48, d = -0.16).

4.2. 14 Electrodes vMMN Results

For the vMMN responses averaged across the 14 electrodes, the mixed-effect ANOVA showed a significant main effect of culture type (F(1, 38) = 4.31, p = .045, η^2_p = .10), while the main effects of cultural relevance and racial essentialism were not significant (Fs(1,38) = 0.24 and 3.72, ps = .63 and .06, η^2_ps = .006 and .089). The two-way interaction effects between racial essentialism and culture type (F(1,38) = 0.39, p = .54, η^2_p = .001), racial essentialism and cultural relevance (F(1,38) = 0.65, p = .80, η^2_p = .002), and between culture type and cultural relevance (F(1,38) = 1.10, p = .30, η^2_p = .028) were not statistically significant. However, most importantly, a significant three-way interaction effect of cultural relevance, culture type, and racial essentialism (F(1,38) = 4.47, p =.041, η^2_p = .11) was observed.

Follow-up repeated measure ANOVAs with the factors cultural relevance and culture type were carried out on the vMMNs for the high and low racial essentialism groups, separately. For the low racial essentialism group, the main effects of culture type and culture relevance were not significant (Fs(1,38) = 1.12 and 0.03, ps= .31 and .88, η^2_ps = .06 and .001), while the two-way interaction effect between culture type and cultural relevance was significant (F(1,38) = 4.72, p = .043, η^2_p = .20). There was no significant difference

between the vMMNs to the Eastern and Western deviants with low culture relevance ($t(19) = 0.88$, $p = .39$, $d = 0.28$). However, the vMMNs to the Eastern deviants with high culture relevance was larger than that of the Western deviants with high culture relevance ($t(19) = -3.15$, $p = .003$, $d = -1.00$).

For the high racial essentialism group, the main effects of culture type, culture relevance, and the two-way interaction between culture type and cultural relevance were not significant (Fs(1,38) = 0.31 to 3.46, ps = .08 to .59, η^2_ps = .016 to .15). No significant differences were found between the vMMNs to the Eastern than the Western deviants with both low and high culture relevance ($ts(19) = -1.46$ and -0.54, $ps = .15$ and .59, $ds = -0.46$ and -0.17).

5. Non-parametric Randomization Test

Alternatives procedures for localizing the vMMN are available. For example, the vMMN can be localized from peak response of the grand average across all conditions. However, this measurement procedure is more appropriate for identifying the peaks of amplitude modulated components in the evoked ERP responses (e.g., increase in N1 amplitude of attended versus unattended conditions). This measurement procedure assumes that the ERP responses to the conditions are different in the amplitudes and has limited tolerance on the latency variations between conditions. As the current study was the first study on vMMNs to cultural deviants, and the latency variation between conditions due to individual differences of racial essentialism were unknown, this procedure may not be appropriate.

To examine whether the results of the current study was produced by the peak vMMN amplitude measurement procedure which could be more sensitive to noise, a non-parametric randomization analysis similar to the simulation procedure in Luck and Gaspelin (2017) was carried out. In this analysis, the racial essentialism group labels (high versus low) were shuffled among the participants, while the cultural relevance (high versus low) and culture type (Eastern versus Western) labels were shuffled among the difference waveforms of different conditions within each participant. The peak mismatch responses were measured from the grand averaged difference waveforms (across participants' waveforms of each condition) using the peak measurement procedure described in the methods session. The main effect and interaction effect contrasts equivalent to the original mixed-effect ANOVA and follow-up tests were calculated. This procedure was repeated 10000 times to generate a null-effect sampling distribution for each main effect and interaction contrasts. The probabilities (*p*-values) of each test were obtained by comparing the contrast measures of the original non-shuffled data with the null-effect sampling distributions. As shown in the Supplementary Table 1 below, the statistical results of the non-parametric randomization tests are similar to that of the original mixed-effect ANOVA and follow-up tests. A similar randomization procedure was used to address the variation in trial number issue (see methods). The results of both randomization tests were similar and led to identical conclusions.

Supplementary Table 1. Comparison of Randomization Test and Mixed-effect Measure ANOVA and Follow-up Analysis Results

	p-value	
**Full Model Analysis**	**Randomization Test**	**Mixed-effect ANOVA**
Culture Type	.038*	0.036*
Cultural Relevance	.36	.58
Racial Essentialism	.033*	.11
Culture Type * Racial Essentialism	.28	.58
Culture Type * Cultural Relevance	.20	.26
Cultural Relevance * Racial Essentialism	.20	.47
Culture Type * Cultural Relevance * Racial Essentialism	**.004***	**.024***

	p-value	
**Follow up analysis by Racial Essentialism**	**Randomization Test**	**Mixed-effect ANOVA**
Low Racial Essentialism:		
Culture Type	.16	.31
Cultural Relevance	.46	.89
Culture Type * Cultural Relevance	**.006***	**.02***
High Racial Essentialism:		
Culture Type	.01*	.045*
Cultural Relevance	.21	.43
Culture Type * Cultural Relevance	**.23**	**.40**

	p-value	
**Pairwise Comparisons: Eastern minus Western Deviants**	**Randomization Test**	**Mixed-effect ANOVA**
Low Racial Essentialism:		
Low Cultural Relevance	.19	.42
High Cultural Relevance	**.007***	**.008***
High Racial Essentialism:		
Low Cultural Relevance	**.007***	**.035***
High Cultural Relevance	.26	.49

Note: *$p < .05$

References

1. Stefanics, G., Kremláček, J. & Czigler, I. Visual mismatch negativity : A predictive coding view. *Front. Hum. Neurosci.* **8**, 1–19 (2014).

2. Kimura, M., Katayama, J. & Murohashi, H. Positive difference in ERPs reflects independent processing of visual changes. *Psychophysiology* **42**, 369–379 (2005).

3. Sel, A., Harding, R. & Tsakiris, M. Electrophysiological correlates of self-specific prediction errors in the human brain. *Neuroimage* **125**, 13–24 (2016).

4. Tse, C.-Y. & Penney, T. B. On the functional role of temporal and frontal cortex activation in passive detection of auditory deviance. *Neuroimage* **41**, 1462–1470 (2008).

5. Tse, C.-Y., Rinne, T., Ng, K. K. & Penney, T. B. The functional role of the frontal cortex in pre-attentive auditory change detection. *Neuroimage* **83**, 870–879 (2013).

6. Tse, C.-Y., Shum, Y. H., Xiao, X. Z. & Wang, Y. Fronto-occipital mismatch responses in pre-attentive detection of visual changes: Implication on a generic brain network underlying Mismatch Negativity (MMN). *Neuroimage* **244**, 118633 (2021).

7. Xiao, X. Z., Wang, Y., Wong, G. C. S., Zhao, K. & Tse, C.-Y. Frontotemporal network in automatic / pre-attentive detection of abstract change: An event-related optical signal (EROS) study. *Neuropsychologia* **164**, (2022).

8. Tse, C.-Y. *et al.* NeuroImage Establishing the functional connectivity of the frontotemporal network in pre-attentive change detection with Transcranial

Magnetic Stimulation and event-related optical signal. *Neuroimage* **179**, 403–413 (2018).

9. Kimura, M., Schröger, E. & Czigler, I. Visual mismatch negativity and its importance in visual cognitive sciences. *Neuroreport* **22**, 669–673 (2011).

10. Pazo-Alvarez, P., Cadaveira, F. & Amenedo, E. MMN in the visual modality: A review. *Biol. Psychol.* **63**, 199–236 (2003).

11. Luck, S. J., & Gaspelin, N. How to get statistically significant effects in any ERP experiment (and why you shouldn't). *Psychophysiology*, **54**, 146-157 (2017).
